# Filarial infection during pregnancy has profound consequences on immune response and disease outcome in children: A birth cohort study

**DOI:** 10.1371/journal.pntd.0006824

**Published:** 2018-09-25

**Authors:** Madhusmita Bal, Manoranjan Ranjit, Ashok K. Satapathy, Hemant K. Khuntia, Sanghamitra Pati

**Affiliations:** ICMR-Regional Medical Research Center, Chandrasekharpur, Bhubaneswar, Odisha, India; University of Zurich, SWITZERLAND

## Abstract

**Background:**

Current Global Program to Eliminate Lymphatic Filariasis (GPELF) that prohibits pregnant mothers and children below two years of age from coverage targeted interruption of transmission after 5–6 rounds of annual mass drug administration (MDA). However, after more than 10 rounds of MDA in India the target has not been achieved, which poses challenge to the researchers and policy makers. Several studies have shown that *in utero* exposure to maternal filarial infections plays certain role in determining the susceptibility and disease outcome in children. But the mechanism of which has not been studied extensively. Therefore the present study was undertaken to understand the mechanism of immune modulation in children born to filarial infected mother in a MDA ongoing area.

**Methodology and principal finding:**

To our knowledge this is the first study to conduct both cellular and humoral immunological assays and follow up the children until older age in a *W bancrofti* endemic area,where the microfilariae (Mf) rate has come down to <1% after 10 rounds of MDA. A total 57 (32: born to infected, 25: born to uninfected mother) children were followed up. The infection status of children was measured by presence of Mf and circulating filarial antigen (CFA) assay. Filaria specific IgG1, IgG2, IgG3 and IgG4 responses were measured by ELISA. Plasma level of IL-10 and IFN-γ were evaluated by using commercially available ELISA kit. The study reveals a high rate of acquisition of filarial infection among the children born to infected mother compared to uninfected mothers. A significantly high level of IgG1 and IgG4 was observed in children born to infected mother, whereas high level of IgG3 was marked in children born to uninfected mother. Significantly high level of IL-10 positively correlated with IgG4 have been observed in infected children born to infected mother, while high level of IFN-γ positively correlated with IgG3 was found in infection free children born to mother free from infection at the time of pregnancy. Moreover a negative correlation between IL-10 and IFN-γ has been observed only among the infected children born to infected mother.

**Significance conclusion:**

The study shows a causal association between maternal filarial infection and impaired or altered immune response in children more susceptible to filarial infection during early childhood. As lymphatic damage that commences in childhood during asymptomatic stage has major implications from public health point of view, understanding maternal programming of the newborn immune system could provide a basis for interventions promoting child health by implementing MDA campaigns towards all women of childbearing age and young children in achieving the target of global elimination of LF.

## Author summary

Lymphatic filariasis (LF) has been targeted for elimination by 2020 through a major global initiative. The elimination strategy mainly aims to interrupt the transmission, or spread of infection, through annual mass drug administration (MDA) to entire at-risk population except the pregnant women and children below two years of age. Nevertheless, evidence exists that maternal *W bancrofti* infection during pregnancy can increase the susceptibility of the offspring to such infection during early childhood, the precise mechanism of which is not clear. Therefore in this study we have made an attempt to elucidate the mechanism of alteration of the foetal immunity due to maternal filarial infection, which may help developing strategy to strengthen the elimination programme. It is known that regulatory T cells are responsible for development of hyporesponsiveness, a condition that supports parasite growth and maternal filarial infection influences the development of T-regulatory cells from infancy to early childhood. Since T-reg cell can induce the production of regulatory cytokine IL-10 that often implicated in induction of IgG4 and we have observed an increased level of IL-10 / IgG4 and decreased levels of IFN- γ/ IgG3 in cord blood of infected mothers, we have thought that the modulation that takes place *in utero* affects the immune response and eventually disease outcome in early childhood. In order to prove our hypothesis, we have followed up a cohort of 57 children born to infected and uninfected mother in a MDA ongoing area of Odisha, India, where we have found a high rate of acquisition of filarial infection by children born to infected mother. Moreover a significantly high as well as a positive correlation between IgG4 and IL-10 levels in children born to infected mother during early childhood indicates that IL-10 and IgG4 contribute to immune modulation that starts during the period of gestation and continues till the early childhood helping the filarial parasites to evade destruction by their host’s immune system. In contrast a high level of IFN-γ and IgG3 in infection free children irrespective of infection status of mother shows the protective mechanism against the parasite. Additionally the correlation between antibodies and cytokines indicates that susceptibility to filarial infection during early childhood is due to the *in utero* modulation of T and B responses. Our findings highlights that every effort should be made to advance implementation policies and continue the focus to use the currently available anti-filarial drugs to prevent filarial infection before pregnancy as well as early childhood to achieve the target on time.

## Introduction

As placental mammals, the pregnant women and the foetus have intense and prolonged interactions during gestation that alters immunity of the new born during their childhood through transferring multiple molecular as well as cellular components [1].With the accumulation of evidences on long-term impact upon responses to helminth and non-helminth antigens, the research on prenatal sensitization or *in utero* exposure has gathered momentum in the field of infectious diseases as it is suspected to expose the neonates to infectious agents during gestation. The immune consequences of parasitic infections can be reflected in the unborn children of infected mothers by generating a number of effects on foetal immune responses [2]. Even though genetic polymorphism, host immune response and environmental factors are known to play pivotal roles towards susceptibility to infection, maternal filarial infection can be considered as one of the risk factors for neonatal infection as evident from our findings [3]. Evidences exist that prenatal sensitization to filarial antigen occurs in human filariasis and that children born from infected mothers are at greater risk of infection relative to offspring of uninfected mothers [4–5]. Over the past few years we have been analysing the influences of maternal filarial infection on neonatal immune response. It appears that trans-placental trafficking of filarial antigens from mother to foetus occurs on a frequent basis [6].Studies have shown that high level of T-regulatory (T-reg) cells at the time of birth and early childhood in children born to infected mother emphasizes that maternal filarial infection influences the development of T-reg cells from infancy to early childhood [7]. Since T- reg cells can induce immunomodulatory cytokine IL-10 that often implicated in induction of immunoregulatory antibody IgG4 and we have observed an increased level of IL-10 / IgG4 and decreased levels of IFN- γ/ IgG3 in cord blood of infected mother [7,8] indicating an initiation of modulation of immune responses in the placenta due to *in utero* priming. Further results of an earlier study has shown that children sensitized to parasite antigens *in utero* produce Th2-dependent specific IgG4 and IgE antibodies at a young age [9]. But the relationship between *in utero* exposure, the development of early immune profiles and disease outcome during early childhood has not been examined comprehensively in populations where filarial infection is endemic. Hence, we have made an attempt to investigate the effect of prenatal sensitization to filarial antigens on filarial specific IgG isotype response, IL-10 level, IFN- γ levels and disease outcome in children. This deserves to be explored because patent filarial infection is now being recognised to occur in much earlier age than previously thought [10, 11] and GPELF excludes the pregnant mothers and children below two years.

## Materials and methods

### Ethics statement

The Human Ethical Committee of the ICMR-Regional Medical Research Centre has approved the study. Children born to healthy pregnant women with or without *W*. *bancrofti* infection were followed up during the present study, while assessing the effect of maternal infection on immune response and disease outcome.All mothers, who have provided face to face oral consent without a signed consent form for participation of self and their children over the entire period of study, have been explained about the purpose of study in local language. The oral consent was preferred because the project involves no risk while giving service to the public and benefits to the ongoing LF elimination programme. The name and detailed address of the participants who have given consent was recorded in our data sheet at the time of enrolment for tracking during follow-up.

### Study design and study participants

This is a birth cohort study conducted in 8 villages of Khurda district-located around 35 km from south of Bhubaneswar, the capital city of Odisha and known to be endemic for filariasis (*W*. *bancrofti*). Detailed information on the methodology and design of the cohort for enrollment of the participants is provided elsewhere [7]. The study subjects were children born to pregnant mothers who were admitted in O&G Department of Khurda District Headquarter Hospital for delivery from July 2009–July 2011.The enrolled children were born full-term, healthy with all baseline information and whose mothers had agreed to continue participation during follow up were included in the study. During the year 2014–2016 the children were followed up in a house-to-house visit. Upon enrolment, mothers completed a questionnaire that queried about age, sex, education and detailed medical (clinical and physical) history, use of prescribed and/or other medications about themselves and their children. Venous blood sample was collected aseptically from each enrolled children under the supervision of a physician and routine laboratory tests (blood group, blood DC (differential count), haemoglobin, total leukocyte count (TLC), microscopic examination of blood smear for parasitic infections) were performed. Treatment was given according to the National Vector Borne Disease Control Programme (NVBDCP) guideline by the physician of local government hospital.

### Detection of Mf and circulating filarial antigen

Infection status of the mother at the time of delivery and children during follow-up was determined by diagnosing the presence of Mf in the peripheral blood collected at night between 20:30 to 22:30 hours. The detection of Mf (*W*. *bancrofti*) was done by microscopy by examining the Giemsa stained thick blood smear. Circulating filarial antigen (CFA) was checked in each serum sample by Og4C3 ELISA test kit (Trop Bio Med, Townsville, Australia) following the instruction of the manufacturer.

### Antigen preparation

Filarial antigen was prepared from adult *Setaria digitata*, a peritoneal dwelling bovine filarial parasite that were collected from a local abattoir in phosphate buffer saline (PBS).Briefly the worms were washed thoroughly with PBS, grounded by means of a mortar and pestle with the addition of PBS followed by 1 min ultrasonication (Artek Systems Corp, Virginia, USA). The homogenate was centrifuged at 10,000 rpm for 20min and the supernatant was collected and stored at −20°C till further use.

### Assessment of filarial specific IgG subclass

The IgG subclasses(IgG1, IgG2, IgG3, and IgG4) to filarial antigen (*Setaria digitata* antigenic extract) were assessed in children born to infected and uninfected mother by enzyme linked immunosorbent assay (ELISA) following the procedure described elsewhere [8]. Plasma samples were tested in duplicates and the mean OD value was calculated. The OD value of a positive control serum, included on all plates, was used to calculate the antibody unit (AU). AU = (OD of test serum/OD of positive control serum) × 100.

### IL-10 cytokine and IFN-γ cytokine analysis

The levels of IL-10 and IFN-γ in plasma was measured using commercially available ELISA kits (E-Bioscience San Diego, CA, USA.) and expressed in pg/mL by interpolation from standard curve as described by manufacturers’ instruction. Briefly, 100 μl of plasma and standards were added to respective well of the antibody coated ELISA plate followed by 50 μl Biotin-conjugate and incubated at room temperature (18 to 25°C) for 2 hours. Wells were washed 3 times with PBST (Phosphate Buffered Saline with 1% Tween 20) and added100 μl of Streptavidin-HRP to each well. After 1 hour of incubation at room temperature and 3 times washing with PBST,100μl of TMB (3,3',5,5'-Tetramethylbenzidine) substrate solution was added to each well followed by incubation at room temperature for 10 minutes. Finally 100 μl of stop solution was added to each well and the optical density (OD) was read at 450 nm using an ELISA plate reader. The detection limit of IL-10 is 1.0 pg/ml and IFN-γ is 0.06 pg/mL.

### Statistical analysis

The statistical analysis was performed using GraphPad Prism software (version 4). Mann-Whitney test was used to analyze the difference between two groups of unpaired data. Fisher's exact test was used to compare the difference of proportions between two groups. The associations between IgG isotype antibodies, IFN-γ and IL-10 levels were analyzed using Spearman’s correlation analysis. The level of significance was set at 0.05.

## Results

**Fig 1:**
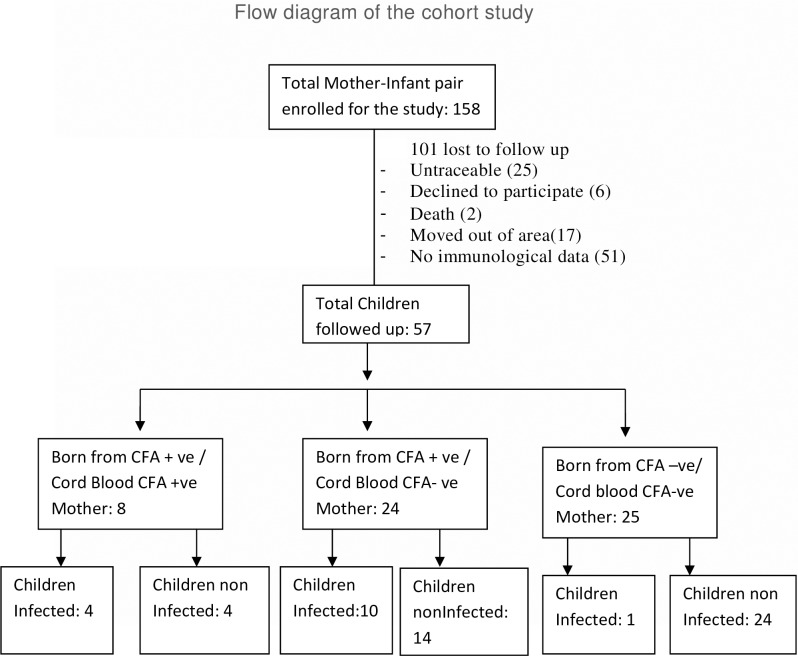
The flow diagram of the cohort study.

An outline of the children included in the present study is reflected in Fig 1.We could involve 57 (Male/Female: 31/ 26) children out of 158 mother-new born pairs enrolled during 2009–2011 and the rest 101 were excluded because they are either non traceable, declined to participate, death of the children, moved out of study area or had no immunological parameter. The age of children at the time of follow-up ranges from 3–8 years and amongst them 26.3% (15/57) were < 5 years. Amongst 57 children included, 32 (Male/Female: 17/15) were born from infected mother and 25 (Male/Female: 14/11) were from uninfected mother. Out of 32 children born to the infected mothers, 14 (43.7%) children have been found to acquire filarial infection and become CFA positive during follow up. No significant difference (p = 0.40) in number of acquiring infection was observed between two age group i.e, < 5 years (5 out of 9) and in 5–8 years of age (9 out of 23). Though at the time of birth 8 out of 32 (25%) children had CFA in their cord blood, 4 of them have acquired infection. In contrast one out of 25 (4.7%) children born to the uninfected mothers has acquired filarial infection and become CFA positive indicating the state of infection during pregnancy as a risk factor for acquiring infection by children born to them [Odds Ratio (OR) = 18.66, 95% CI: 2.2432 to 155.334, Z = 2.70, p = 0.0006]. However, neither Mf in peripheral blood nor any clinical signs/symptoms of filariasis was detected in any of the children born to infected and/or uninfected mothers (Table 1).

**Table 1:** Characteristic of children born to infected and uninfected mother during follow-up in a MDA ongoing area of Odisha, India.

Children	Status of mother at the time of delivery		
	Infected	Uninfected	P value
Total children followed up	32	25	
Male(%)	17(53.1)	14(56)	P = 0.75
Female(%)	15(46.8)	11(44)	P = 0.83
Age groups(years) <5(%) 5-8(%)	9(28.1) 23(71.9)	6(24.0) 19(76.0)	P = 0.72 P = 0.72
Rural(%) Urban(%)	29(87.8) 3(9.3)	21(84.0) 4(16.0)	P = 0.683 P = 0.447
Presence of Mf in cord blood	0	0	
Presence of CFA in cord blood(%)	8(25)	0	P = 0.0075
Filarial infection present in children(%)	14(43.7)	1(4)	P = 0.0006
Clinical symptoms	No	No	

For further analysis the eligible subjects were categorised into 4 groups based on the infection status of mother during enrolment and presence/ absence of CFA in children during follow up *i*.*e*. Group I: CFA positive children born to infected mother (M^+^Ch^+^, n = 14), Group II: CFA negative children born to infected mother (M^+^Ch^-^, n = 18), Group III: CFA negative children born to uninfected mother (M^-^Ch^-^, n = 24) and Group IV: CFA positive children born to uninfected mother (M^-^Ch^+^, n = 1)

**Fig 2:**
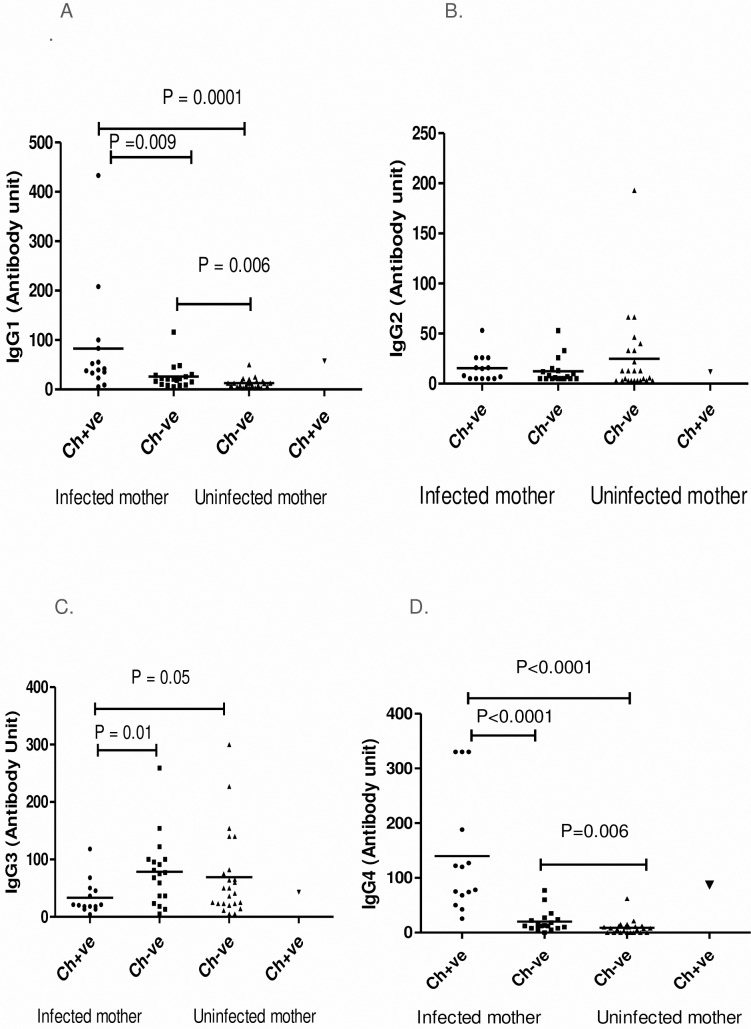
Filaria-specific IgG isotype antibody responses in children during follow-up. Antibody units of filaria-specific IgG1 (A), IgG2 (B), IgG3(C) and IgG4 (D) response in children born from filarial infected and uninfected mothers during follow-up study. Each dot represents an individual's antibody levels and lines represent the mean values. Ch+ve: children were CFA positive; Ch−ve: children CFA negative.

**Fig 3:**
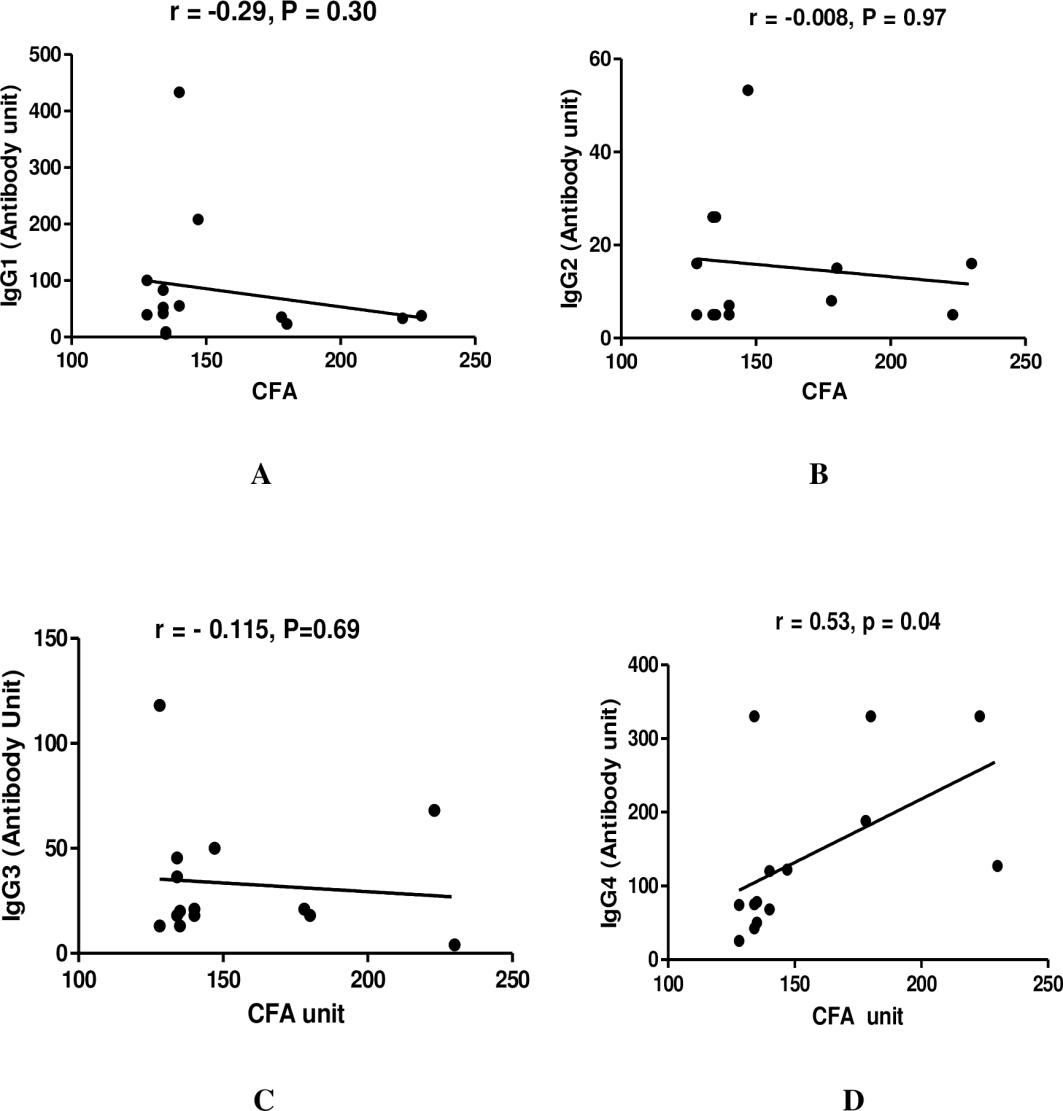
The relationship between IgG antibody isotypes with circulating filarial antigen (CFA) in infected children (n = 14) born to infected mother during the follow-up study. Each dot represents value from a single subject while the solid lines represent the regression lines. Values of P and r derive from Spearman’s correlation analysis. (A) IgG1 vs CFA (r = -0.29, p = 0.30) (B) IgG2 vs CFA (r = -0.008, p = 0.97) (C) IgG3 vs CFA (r = - 0.115, p = 0.69) (D) IgG4 vs CFA (r = 0.53, p = 0.04).

To evaluate the impact of maternal infection during pregnancy on development of IgG isotypes in children during their early childhood, we have analyzed the IgG isotypes in children born to infected and infection free mother during follow up.The IgG1 and IgG4 antibody level in children born to infected mother (M^+^Ch^+^, M^+^Ch^-^) were significantly high (IgG1: P<0.001 for M^+^Ch^+^ and P = 0.006 for M^+^Ch^-^; IgG4: P <0.001 for M^+^Ch^+^ and P = 0.006 for M^+^Ch^-^) compared to children born to uninfected mother. However a significant difference in IgG1 and IgG4 levels (P = 0.009 for IgG1, and p <0.0001 for IgG4) was observed among CFA +ve and CFA–ve children born to infected mother. Whereas a significantly high level of IgG3 levels were observed in CFA–ve children (M^+^Ch^-^, P = 0.01 and M^-^Ch^-^, P = 0.05) compared to CFA positive children. On comparison no difference was observed in IgG3 level among infection free children born to infected as well as uninfected mother. Analysing the level of IgG2 in different groups no difference was observed in infected and uninfected children (Fig 2).But while analysing the association of antibody isotypes with CFA units, a positive correlation was only observed between IgG4 and CFA units in infected children born to infected mothers (r = 0.53, p = 0.04) as depicted in Fig 3 (D).

**Fig 4:**
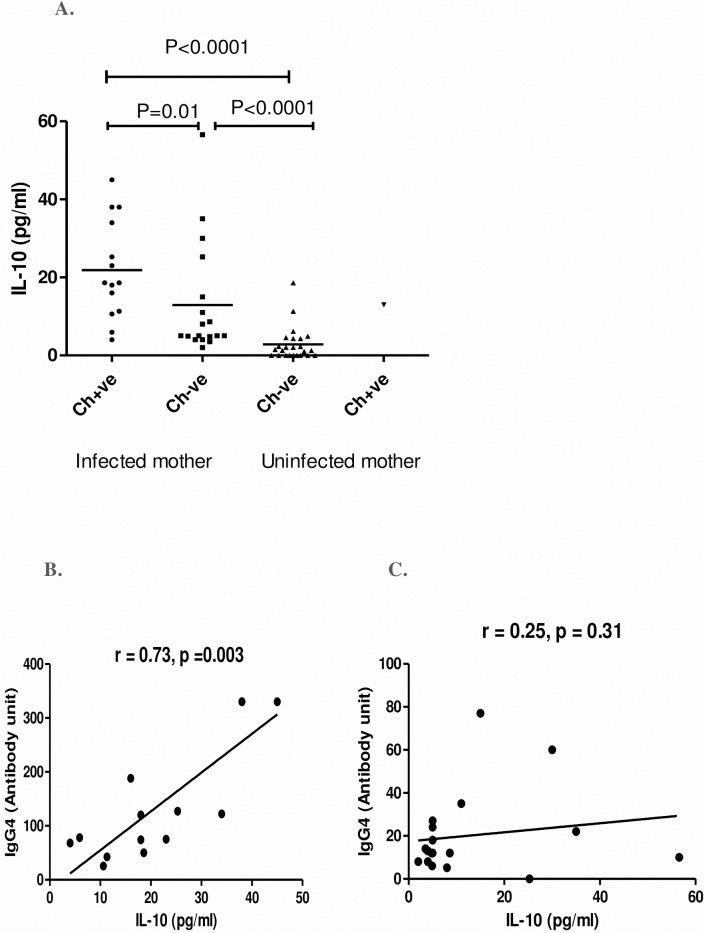
Plasma level of IL-10 and their correlation with IgG4 in CFA +ve and CFA-ve children born from infected and uninfected mother at the time of follow-up. (A) Each dot represents an individual's IL-10 level and lines represent the mean values. Ch+ve: children were CFA positive; Ch−ve: children CFA negative. Correlation between IL-10 and IgG4 in CFA +ve children (n = 14, B) and CFA–ve children (n = 18, C) born to infected mother. Each dot represents value from a single subject while the solid lines represent the regression lines. Values of p and r derive from Spearman’s correlation analysis.

We have quantitatively assessed the level of IL10, the hallmark cytokine for regulatory response, in children born to infected and uninfected mothers to evaluate the role of differentiated T helper cell subsets in filarial infection. Quantitative assessment of IL-10 indicates that children born to infected mother have significantly high level of plasma IL-10 compared to children born to uninfected mother (M^+^Ch^+^ vs M^-^Ch^-^, P < 0.001 and (M^+^C^-^ vs M^-^Ch^-^, P<0.001). However, significantly high (P = 0.01) level of IL-10 was observed in children who have acquired infection than those free from infection even born to infected mother (Fig 4A). A significant positive correlation (r = 0.73, p = 0.003) between IL-10 and IgG4 level was observed in infected children born to infected mother (Fig 4B) but no such correlation (r = 0.25, p = 0.31) was found in infection free children born to infected mother (Fig 4C).

**Fig 5:**
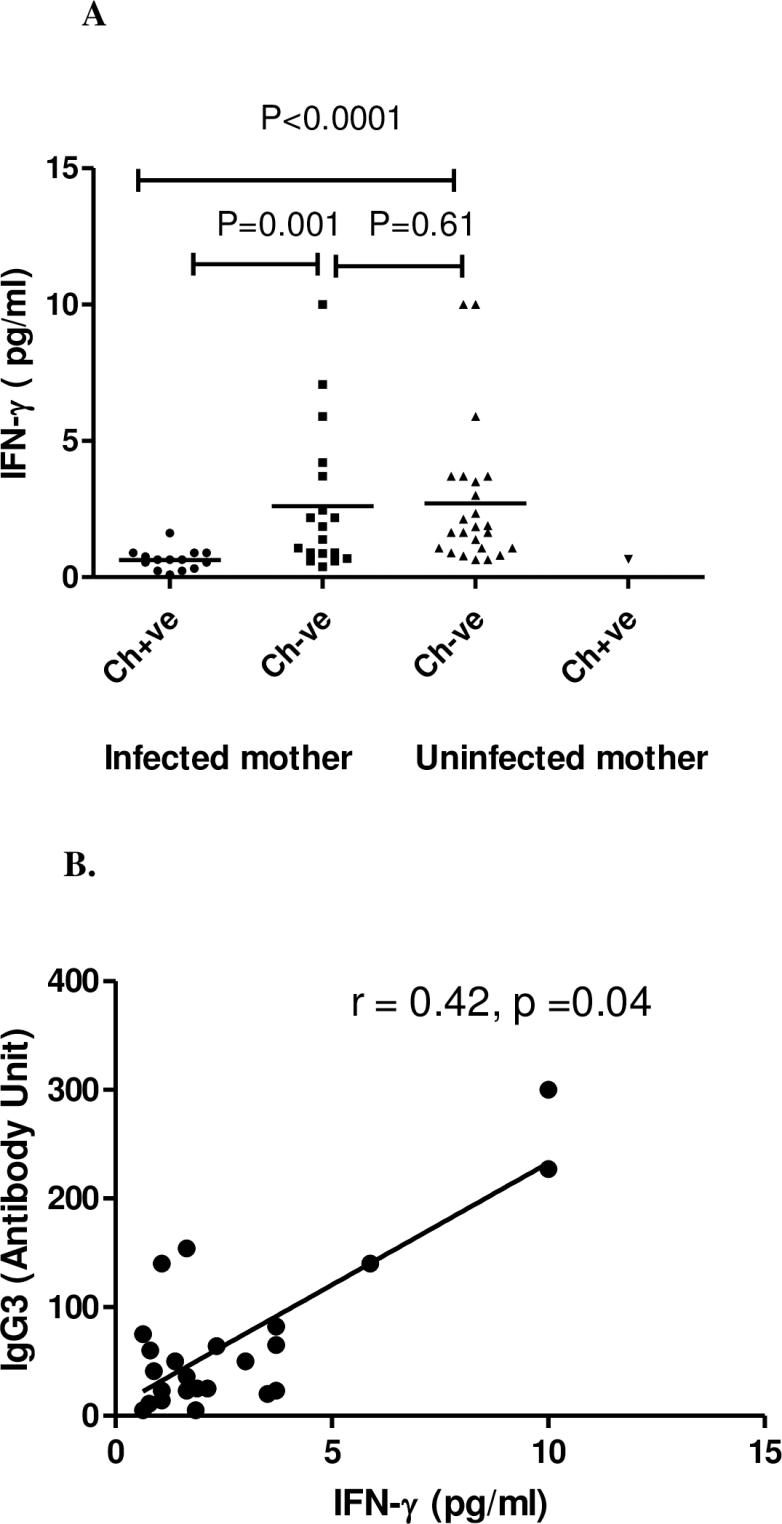
Plasma level of IFN-v and their correlation with filarial specific IgG3 in CFA +ve and CFA-ve children born from infected and uninfected mother at the time of follow-up. (A) Each dot represents an individual's IFN-γ level and lines represent the mean values. Ch+ve: children were CFA positive; Ch−ve: children CFA negative. (B) Correlation between IFN-γ and IgG3 in CFA–ve children (n = 24) born to infection free mother. Each dot represents value from a single subject while the solid lines represent the regression lines. Values of p and r derive from Spearman’s correlation analysis.

To assess the modulation of differentiated T helper cell subsets by filarial infection, IFN-γ (Th1) was evaluated in children during follow up. The plasma level of IFN-γ was observed to be significantly high in CFA negative children born to mother irrespective of their infection status at the time of enrolment than CFA +ve children (M^+^Ch^-^ vs M^+^Ch^+^: P = 0.001 and M^-^Ch^-^ vs M^+^Ch^+^, P< 0.0001). But no significant difference was observed among the infection free children born to either infected or uninfected mother (Fig 5A).However when a correlation was drawn between IFN-γ and IgG3, a positive correlation (r = 0.42, p = 0.04)was observed only in CFA–ve children born to infection free mothers (Fig 5B).

**Fig 6:**
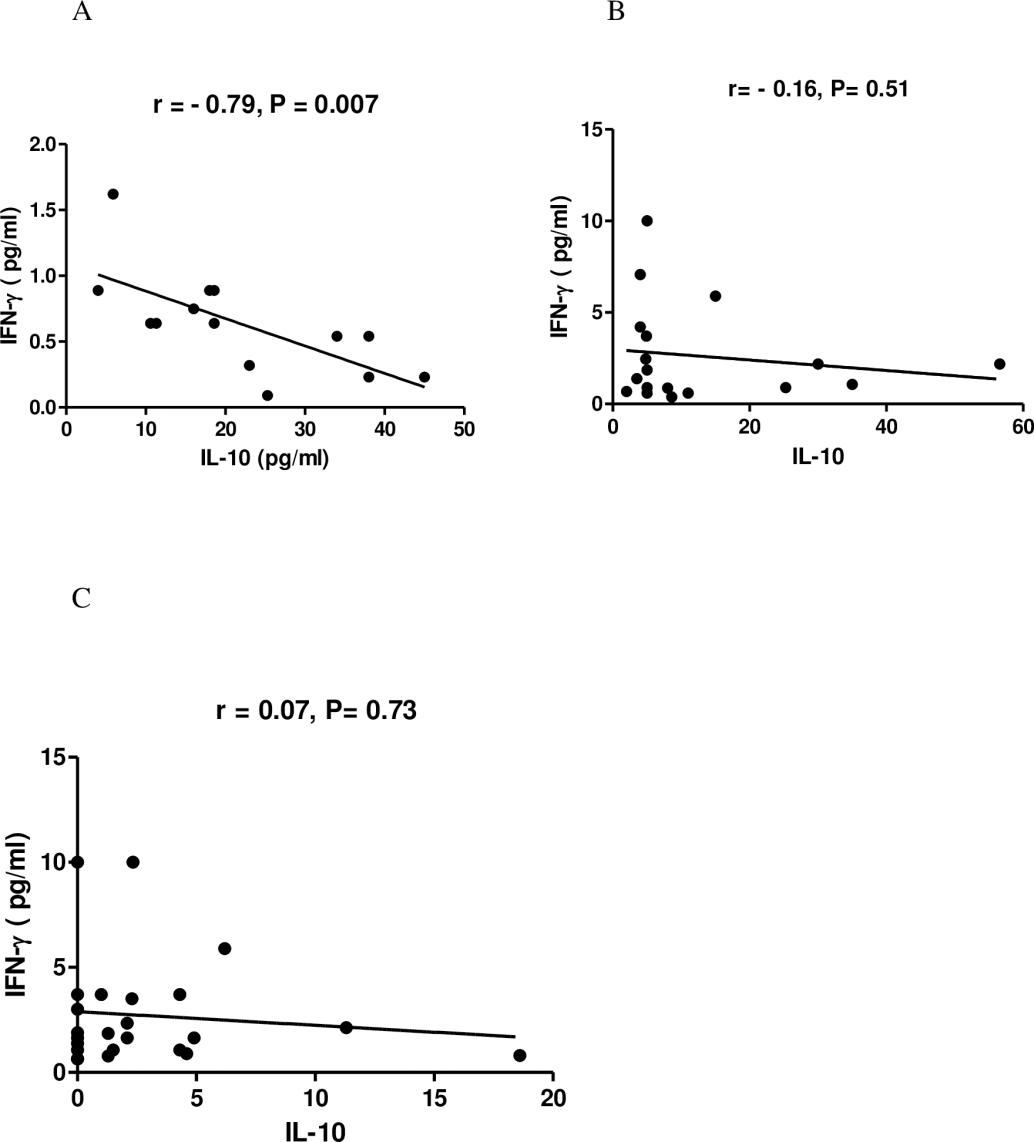
The relationship between regulatory cytokine IL-10 with IFN-γ level of CFA +ve and CFA–ve children born to infected and uninfected mother during the follow-up study. Each dot represents value from a single subject while the solid lines represent the regression lines. Values of P and r derive from Spearman’s correlation analysis. (A) CFA+ve children from infected mother (n = 14, r = -0.79, p = 0.007) (B) CFA–ve children from infected mother (n = 18, r = -0.16, p = 0.51) (C) CFA negative children of uninfected mother (n = 24, r = 0.07, p = 0.73).

To find out the effect of maternal infection on regulation of Th1 (IFN-γ)and Th2 (IL-10) type of cytokine in children during their natural exposure to filarial infection in a MDA ongoing area, a correlation was made between IFN-γ levels and IL-10 level in children during the follow up. As shown in Fig 6 a highly significant negative correlation was observed among CFA positive children born to infected mother (r = -0.79, p = 0.007; Fig 6A**).** However no correlation between IL-10 and IFN-γ was observed among the CFA negative children born to neither infected nor uninfected mother (Fig 6B and 6C).

## Discussion

Our study demonstrates that maternal filarial infection during pregnancy has profound consequences on IgG isotypes as well as cytokine profiles (IFN-γ and IL-10) in early life of children born to infected and uninfected mothers residing in a MDA ongoing area. Further a correlation between IgG4 vs IL-10, IgG3 vs IFN-γ and IL-10 vs IFN-γ signifies that the immunomodulatory action started due to *in utero* priming ultimately determine the disease outcome during early childhood.

The transfer of maternal immunity and programming of the newborn immune system has long been a debate in field of infectious diseases. During the present study the high level of filaria-specific IgG4 in children born from infected mothers as well as cord blood as shown in our previous study exhibits that *in utero* priming to filarial antigens can induce non-complement-fixing immunoglobulin (IgG4) which unable to activate any of the protective immune mechanisms that involve complement as well as antibody dependent cell-mediated cytotoxicity [8, 12]. Further role of regulatory T cells in modulation of human immune response and induction of IgG4 production in helminthic infection that promotes parasite tolerance highlights its importance in immunity to infectious agents [13].High expression of T-reg cells in children and cord blood of infected mother as evident in our earlier study[7] and elevated level of IgG4 in infected children born to infected mother in the present study reveals that increasing susceptibility to infection might be due to *in utero* modification of foetal immune responses towards the production immunoregulatory antibody (IgG4) that helps for survival of the parasite. In addition absence of clinical symptoms in infected children during follow up might be because of high plasma concentration of IgG4 as evident by others in asymptomatic filarial infection. Whereas the positive correlation between CFA and IgG4 in infected children might be associated with the worm loads [12].On the contrary high level of filaria-specific IgG3 and low level of IgG4 in children from uninfected mother stress that in absence of immunoregulation, the host’s immunocompetent antigen-presenting cells activate effector T-cells, which in turn induce B cells to produce cytolytic antibodies (IgG3) that kills the parasite. Our prediction of protective role of IgG3 is based on the reports of earlier authors who have described an inverse relationship between presence of IgG3 and absence of active filarial infection [14] and efficacy of IgG3 to neutralize pathogens at the port of entry into the body via FcγR-mediated effector responses [15]. Association of malaria and/or helminthic infection during pregnancy that has been found to impair IgG antibody responses to key protective antigens of Hib and diphtheria in infants born from infected mothers like the present study may raise question to the overall success of global control programme [2].

Filarial parasite antigen-specific T cell hypo-responsiveness is mediated particularly by IL-10 and TGF- β regulatory cytokines, which is characterized by a modified Th2 response associated with increased frequencies of IL-10 producing CD4 T cells that act on B cell to induce production of IgG4 [16]. In the present study the high level of IL-10 along with its positive correlation with IgG4 in infected children reveals that the regulatory cytokine is directly suppressing the immune responses during patent filarial infection. The elevated level of IL-10 and IgG4 in the cord blood of infected mother at the time of delivery as well as in early childhood emphasizes that IL-10 indirectly regulate not only the antibody response to filarial antigens but also the function of antigen presenting cells [8, 16] making the children more susceptible to filarial infection during their early exposure. The high level of IL-10 among infected pregnant mother at the time of delivery and high IL-10 level in infected children during follow up unveils that maternal cytokine during pregnancy, which could be a proxy for child’s environmental factors, has highest impact at early age of the children with no or little influence from genetic factors [17]. Like ours other workers have also observed the development of strong Th2 responses in children born in areas endemic for helminthic infections that increase with age during early childhood [18].Whereas association of infection free status of children during follow up irrespective of infection status of mother with increased IFN-γ (hallmark cytokines for Th1) production might be due to the maturation of child’s immune system that has progressed well and thereby promoting the Th1 (IFN-γ) response, because production of Th1-type cytokine at early age is independently associated with maternal cytokine and gene polymorphisms [17, 19]. Moreover a significant positive correlation between IFN - γ and IgG3 only in infection free children born to uninfected mothers could be due to high expression of IgG3 and IFN-γ in *utero* as compared to children of infected mother [8]. This might be the cause for such discrepancy in correlation between IgG3 and IFN- γ level although infection free children born to infected mother have substantial amount of IgG3 and IFN- γ level. Further, maternal filarial infection increases childhood susceptibility to *W*. *bancrofti* and skews filaria-specific immunity toward a Th2-type cytokine response [4]. Here we can speculate that susceptibility to filarial infection may not strictly be due to the expression of filarial specific immune responses but may be a function of the cytokine environment in which the parasite survives and develops [20]. But diminished IFN-γ response in children born to infected mother has been predicted to bias the immune response towards development of T regulatory and Th2 responses rather than Th1 responses [7].Moreover a negative correlation between IFN-γ and IL-10 observed among the infected children born to infected mother highlights epigenetic changes within the naïve T cell compartment affecting Th2 and Th1 cell differentiation as described by others in offspring of mothers with chronic helminthic infection [21, 22]

The high incidence of filarial infection in children born to infected mothers in our study could be due to alteration in the immunity of the new born during their childhood because of prolonged interaction during gestation [1, 6, 23] and apparently not because of the differences in exposure to mosquito-borne infective stage larvae as suggested by some [24, 25] since the children included in the study are living in the same endemic area with equal chance of exposure to active transmission. However absence of visible clinical manifestation in the childhood as observed in this study does not mean that they are free from disease since recent studies have shown damage to the lymphatic system by the parasites in children that remains subclinical for years, before manifestation of clinical features of the adult disease syndromes [11].This type of lymphatic damage commencing in childhood has major implications for prevention of disease in individual patients and for the broader public health efforts to overcome all childhood illnesses [26]. Therefore community-based control programs for filarial infection should target women of childbearing age for treatment that ultimately can reduce the morbidity in children substantially due to filariasis after birth.

The obvious limitations of the current study is small sample size corresponding to both children born to infected and uninfected mothers. Further only one child born from uninfected mother having filarial infection prohibit us for comparing with other groups and draw a correlation between cytokine profile and IgG isotypes. Nevertheless, although important to gather data on IgG isotype levels and cytokine profile in all four groups, the primary objective of our study was to assess immunomodulation took place in relation to acquisition of disease in children born to infected and uninfected mother.

In spite of these limitations the study divulge that increased level of IL-10 and IgG4 could initiate a cascade of hyporesponsive mechanism from infancy to early childhood making the children born to infected mother more susceptible to filarial infection. Whereas high level of IFN-γ and IgG3 level irrespective of the infection status of mother in children through different effector mechanisms gives protection. Thus susceptibility to filarial infection during early childhood is not simply due to immaturity of the immune system but how immune homeostasis is regulated through modulation of T and B cell response since birth. Overall, our observations reinforce the importance of filarial treatment and targeted prevention of filarial infections in young children as well as women of childbearing age. To prevent the prenatal immune priming and tolerance the current MDA must consider ensuring coverage of all women of childbearing age before pregnancy so as to make them free from infection and children, to reduce the chance of sub clinical lymphatic damage in order to achieve the goal of GPELF.

## Supporting information

S1 Checklist STROBE statement and checklist for this study. (DOC)

S1 Fig Correlation between IFN-γ with filarial specific IgG3 in infected (A) and infection free (B) children born to infected mother.
(TIF)
