# Supplementary material for: Filarial infection during pregnancy has profound consequences on immune response and disease outcome in children: A birth cohort study
Source: PLoS Negl Trop Dis. 2018 Sep 25;12(9):e0006824. doi: 10.1371/journal.pntd.0006824 (PMC6173457; doi:10.1371/journal.pntd.0006824)
Supplement: S1 Checklist — (DOC) [file pntd.0006824.s001.doc]

STROBE Statement—Checklist of items that should be included in reports of ***cohort studies***

|  | Item No | Recommendation |
| --- | --- | --- |
| **Title and abstract** | 1 | (*a* Filarial infection during pregnancy has profound consequences on immune response and disease outcome in children: a birth cohort study |
|  |  | (*b* The present study was undertaken to understand the mechanism of immune modulation takes place in children born to filarial infected and uninfected mother in a MDA ongoing area. This birth cohort study was conducted in 9 villages of Khurda district of Odisha known to be endemic for lymphatic filariasis and where the microfilariae (Mf) rate has come down to <1% after 10 rounds of annual mass drug administration (MDA). A total 57 (32: born to infected, 25: born to uninfected mother) children were followed up. The study reveals a high rate of acquisition of filarial infection among the children born to infected mother compared to uninfected mothers. A significantly high level of IgG1 and IgG4 was observed in children born to infected mother, whereas high level of IgG3 was marked in children born to infection free mother. Significantly high levels of IL-10 and a positive correlation with IgG4 have been observed in infected children born to infected mother, while high level of IFN-γ and its positive correlation with IgG3 was found in infection free children born to mother free from infection at the time of pregnancy. Moreover a negative correlation between IL-10and IFN-γ has been observed only among the infected children born to infected mother. The study shows a causal association between maternal filarial infection and impaired or altered immune response in children making them more susceptible to filarial infection in early life. As lymphatic damage commencing in childhood during asymptomatic stage has major implications on public health point of view, understanding maternal programming of the newborn immune system could provide a basis for interventions promoting child health by implementing MDA campaigns towards all women of childbearing age and young children in achieving the target of global elimination of LF. |
| Introduction | | |
| Background/rationale | 2 | Current Global Program to Eliminate Lymphatic Filariasis (GPELF) that prohibits pregnant mothers and children below two years of age from coverage targeted interruption of transmission after 5-6 rounds of annual MDA. However, after more than 10 rounds of MDA in India the target has not been achieved, which poses challenge to the researchers and policy makers. Several studies have shown that in utero exposure to maternal filarial infections plays certain role in determining the susceptibility and disease outcome in children. But the mechanism of which has not been studied extensively. Therefore the present study was undertaken to understand the mechanism of immune modulation in children born to filarial infected mother in a MDA ongoing area |
| Objectives | 3 | The transfer of maternal immunity and programming of the newborn immune system has long been a debate in field of infectious diseases. Evidences exist that prenatal sensitization to filarial antigen occurs in human filariasis and that children born from infected mothers are at greater risk of infection relative to offspring of uninfected mother. But the mechanism is not exactly known. In our earlier study we have shown that maternal filarial infection influences the development of T-regulatory cells from infancy to early childhood. Since T reg cell can induce immunomodulatory cytokine IL-10 that often implicated in induction of immuniregulatory antibody IgG4 and we have observed an increased level of IL-10 / IgG4 and decreased levels of IFN- γ/ IgG3 in cord blood of infected mother indicating an initiation of immune modulation at cord blood due to in utero priming. In the present study We have made an attempt to investigate the effect of prenatal sensitization to filarial antigens on filarial specific IgG isotype response, IL-10 level, IFN- γ levels and disease outcome in children. |
| Methods | | |
| Study design | 4 | This is a birth cohort study. |
| Setting | 5 | Children born to the mothers who were admitted in O&G Department of Khurda District Headquarter Hospital, Odisha(India) for delivery from July 2009–July 2011 and are permanent residents of the filariae endemic area have been enrolled for the study conveniently. During the year 2014 -2016 the children were followed up in a house-to-house visit. The detailed clinical history was collected in a standardized questionnaire, venous blood sample was collected aseptically from each enrolled children under the supervision of a physician |
| Participants | 6 | All mothers, who have provided face to face oral consent for participation of self and their children over the entire period of study were included in the study. The name and detailed address of the participants who have given consent was recorded in our data sheet at the time of enrolment for tracking during follow-up. During this study we have followed up a cohort of 57 children (32 from infected mother and 25 from uninfected mother) in a MDA ongoing area of Odisha, India. |
|  |  |  |
| Variables | 7 | i) Mf / CFA status of children during follow up, Diagnosis of Mf was done in thick blood smear of peripheral blood collected at night between 20:30 and 22:30 by microscopy and detection of CFA in serum samples using commercial Og4C3 antigen detection assay(ii) ) IgG isotypes, IL-10 and IFN-γ were measure by ELISA . |
| Data sources/ measurement | 8* | The data generated by examination of blood collected directly from the children during night blood survey. Diagnosis of Mf was done in thick blood smear of peripheral blood collected at night between 20:30 and 22:30 by microscopy and detection of CFA in serum samples using commercial Og4C3 antigen detection assay.  The IgG subclasses(IgG1, IgG2, IgG3, and IgG4) to filarial antigen (*Setaria digitata* antigenic extract) were assessed in children born to infected and uninfected mother by enzyme linked immunosorbent assay (ELISA). The levels of IL-10 and IFN-γ in plasma was measured using commercially available ELISA kits (E-Bioscience San Diego, CA, USA.) and expressed in pg/mL by interpolation from standard curve as described by manufacturers’ instruction. |
| Bias | 9 | Only healthy children born to mothers who were admitted in the hospital during delivery and are the permanent residents of the study area has been enrolled to avoid any sampling bias. |
| Study size | 10 | The study size was arrived basing on the collection of blood sample from eligible children whose mothers had agreed to give. Also it depends on the immunological data available with us . |
| Quantitative variables | 11 | Quantitative variables were analysed using appropriate parametric tests |
| Statistical methods | 12 | The statistical analysis was performed using GraphPad Prism software (version 4). Mann-Whitney test was used to analyze the difference between two groups of unpaired data. Fisher's exact test was used to compare the difference of proportions between two groups. The associations between IgG isotype antibodies, IFN-γ and IL-10 levels were analyzed using Pearson's correlation analysis. The level of significance was set at 0.05. |
|  |  |  |
|  |  |  |
|  |  |  |
|  |  |  |
| Results | | |
| Participants | 13* | We could involve 57 (Male/Female: 31/26 ) children out of 158 mother-new born pairs enrolled during 2009 -2011 and rest 101excluded because they are either non traceable, decline to participate, death of the children, moved out of study area or non availability of immunological parameter. Amongst 57 children included, 32 (Male/Female: 17/15) were born from infected mother and 25 (Male/Female: 14/11) were from uninfected mother |
|  |  | (b) Give reasons for non-participation at each stage  Non-participation was due to non traceable, decline to participate, death of the children, moved out of study area or non availability of immunological parameter |
|  |  | (c) Consider use of a flow diagram |
| Descriptive data | 14* | The children during follow up belongs age range of 4 -8 years and majority of them belongs to rural area. However, neither Mf in peripheral blood nor any clinical signs/symptoms of filariasis was detected in any of the children born to infected and/or uninfected mothers. Out of 32 children born to the infected mothers, 14 (43.7%) children have been found to acquire filarial infection and become CFA positive during follow up. Though at the time of birth 8 out of 32 (25%) children had CFA in their cord blood, 4 of them have acquired infection. In contrast one out of 25 ( 4.7%) children born to the uninfected mothers has acquired filarial infection and become CFA positive indicating the state of infection during pregnancy as a risk factor for acquiring infection by children born to them (OR = 18.66, 95%CI: 2.2432 to 155.334, Z = 2.70, p = 0.0006). |
|  |  |  |
|  |  |  |
| Outcome data | 15* | The study reveals a high rate of acquisition of filarial infection among the children(14/32) born to infected mother compared to uninfected mothers (1/25). A significantly high level of IgG1 and IgG4 was observed in children born to infected mother, whereas high level of IgG3 was marked in children born to infection free mother. Significantly high levels of IL-10 and a positive correlation with IgG4 have been observed in infected children born to infected mother, while high level of IFN-γ and its positive correlation with IgG3 was found in infection free children born to mother free from infection at the time of pregnancy. Moreover a negative correlation between IL-10and IFN-γ has been observed only among the infected children born to infected mother. |
| Main results | 16 | Amongst 57 children included, 32 (Male/Female: 17/15) were born from infected mother and 25 (Male/Female: 14/11) were from uninfected mother. Out of 32 children born to the infected mothers, 14 (43.7%) children have been found to acquire filarial infection and become CFA positive during follow up. Though at the time of birth 8 out of 32 (25%) children had CFA in their cord blood, 4 of them have acquired infection. In contrast one out of 25 ( 4.7%) children born to the uninfected mothers has acquired filarial infection and become CFA positive indicating the state of infection during pregnancy as a risk factor for acquiring infection by children born to them (OR = 18.66, 95%CI: 2.2432 to 155.334, Z = 2.70, p = 0.0006). However, neither Mf in peripheral blood nor any clinical signs/symptoms of filariasis was detected in any of the children born to infected and/or uninfected mothers. TheIgG1 and IgG4 antibody level in children born to infected mother (M^+^Ch^+^, M^+^Ch^-^) were significantly high (IgG1 : P=0.001 for M^+^Ch^+^ and P=0.006 for M^+^Ch^-^ ; IgG4 : P <0.001 for M^+^Ch^+^ and P=0.006 for M^+^Ch^-^) compared to children born to uninfected mother (M^-^Ch^-^) irrespective of infection status. However a significant difference in IgG1 and IgG4 levels (P =0.009 for IgG1, and p <0.0001 for IgG4) was observed among CFA +ve and CFA –ve children born to infected mother. Whereas a significantly high level of IgG3 levels were observed in CFA –vechildren (M^+^Ch^-^, P = 0.01 and M^-^Ch^-^, P = 0.05) compared to CFA positive children (M^+^Ch^+^) irrespective of infection status of mother at the time of enrolment. On comparison no difference was observed in IgG3 level among infection free children born to infected as well as uninfected mother. Analysing the level of IgG2 in different groups no difference was observed in infected and uninfected children. A positive correlation was only observed between IgG4 and CFAunits in infected children born to infected mothers (r = 0.53, p = 0.04). . Quantitative assessment of IL-10 indicates that children born to infected mother have significantly high level of plasma IL-10 compared to children born to uninfected mother (M^+^Ch^+^vs M^-^Ch^-^ , P < 0.001 and (M^+^C^-^vs M^-^Ch^-^, P<0.001). However significantly high (P=0.01) level of IL-10 was observed in children those have acquired infection than those free from infection even born to infected mother. A significant positive correlation (r = 0.823, p = 0.003) between IL-10 and IgG4 level was observed in infected children born to infected mother but no such correlation (r = 0.15, p = 0.53) was found in infection free children born to infected mother. The plasma level of IFN-γ was observed to be significantly high in CFA negative children born to mother irrespective of their infection status at the time of enrolment than CFA +ve children (M^+^Ch^-^vsM^+^Ch^+^ : P=0.001 and M^-^Ch^-^ vs M^+^Ch^+^, P< 0.0001). But no significant difference was observed among the infection free children born to either infected or uninfected mother. However when a correlation was drawn between and IFN-γ and IgG3, a positive correlation ( r = 0.77, p <0.001)was observed only in CFA –ve children born to infection free mothers. a highly significant negative correlation was observed among CFA positive children born to infected mother (p = 0.006, r= -0.688**).**However no correlation between IL-10 and IFN-γ was observed among the CFA negative children born to neither infected nor uninfected mother. |
|  |  |  |
|  |  |  |
| Other analyses | 17 | .No other analysis has been done |
| Discussion | | |
| Key results | 18 | The study divulge that increased level of IL-10 and IgG4 may initiate a cascade of hyporesponsive mechanism from infancy to early childhood making the children born to infected mother more susceptible to filarial infection. Whereas high level of IFN-γ and IgG3 level irrespective of the infection status of mother in children through different effector mechanisms gives protection |
| Limitations | 19 | Regarding the limitations of the current study is small sample size corresponding to both children born to infected and uninfected mothers. Further only one children born from uninfected mother having filarial infection prohibit us for comparing with other groups and draw a correlation between cytokine profile and IgG isotypes. Nevertheless, although important to gather data on IgG isotype levels and cytokine profile in all four groups, the primary objective of our study was to assess immunomodulation took place in relation to acquisition of disease in children born to infected and uninfected mother. |
| Interpretation | 20 | High rate of infection among the children born to mothers infected with *W bancrofti* during pregnancy might be one of the reasons for persistence of filarial infection among the children < 5 years even though Mf rate has come down below threshold level(<1%) in this endemic area after implementation of 10 rounds of MDA. The absence of symptom in children does not mean that they are free from infection because damage to the lymphatic system by the parasites occurs much earlier that remains subclinical for years, before manifestation of clinical features of the adult disease syndromes. This type of lymphatic damage commencing in childhood has major implications for prevention of disease in individual patients and for the broader public health efforts to overcome all childhood illness. So understanding maternal programming of the newborn immune system could provide a basis for interventions promoting child health by implementing MDA campaigns towards all women of childbearing age and young children in achieving the target of global elimination of LF |
| Generalisability | 21 | The results can be generalized beyond the immediate study since many follow up studies carried out in other helminthic infection in all over the world have shown that maternal infection has a profound consequence on immune response of children and ultimately on disease outcome. |
| Other information | | |
| Funding | 22 | No specific funding was allotted to this study. It was supported by institutional funding. |

*Give information separately for exposed and unexposed groups.

**Note:** An Explanation and Elaboration article discusses each checklist item and gives methodological background and published examples of transparent reporting. The STROBE checklist is best used in conjunction with this article (freely available on the Web sites of PLoS Medicine at http://www.plosmedicine.org/, Annals of Internal Medicine at http://www.annals.org/, and Epidemiology at http://www.epidem.com/). Information on the STROBE Initiative is available at http://www.strobe-statement.org.
