# Supplementary figures and images for: Filarial infection during pregnancy has profound consequences on immune response and disease outcome in children: A birth cohort study
Source: PLoS Negl Trop Dis. 2018 Sep 25;12(9):e0006824. doi: 10.1371/journal.pntd.0006824 (PMC6173457; doi:10.1371/journal.pntd.0006824)

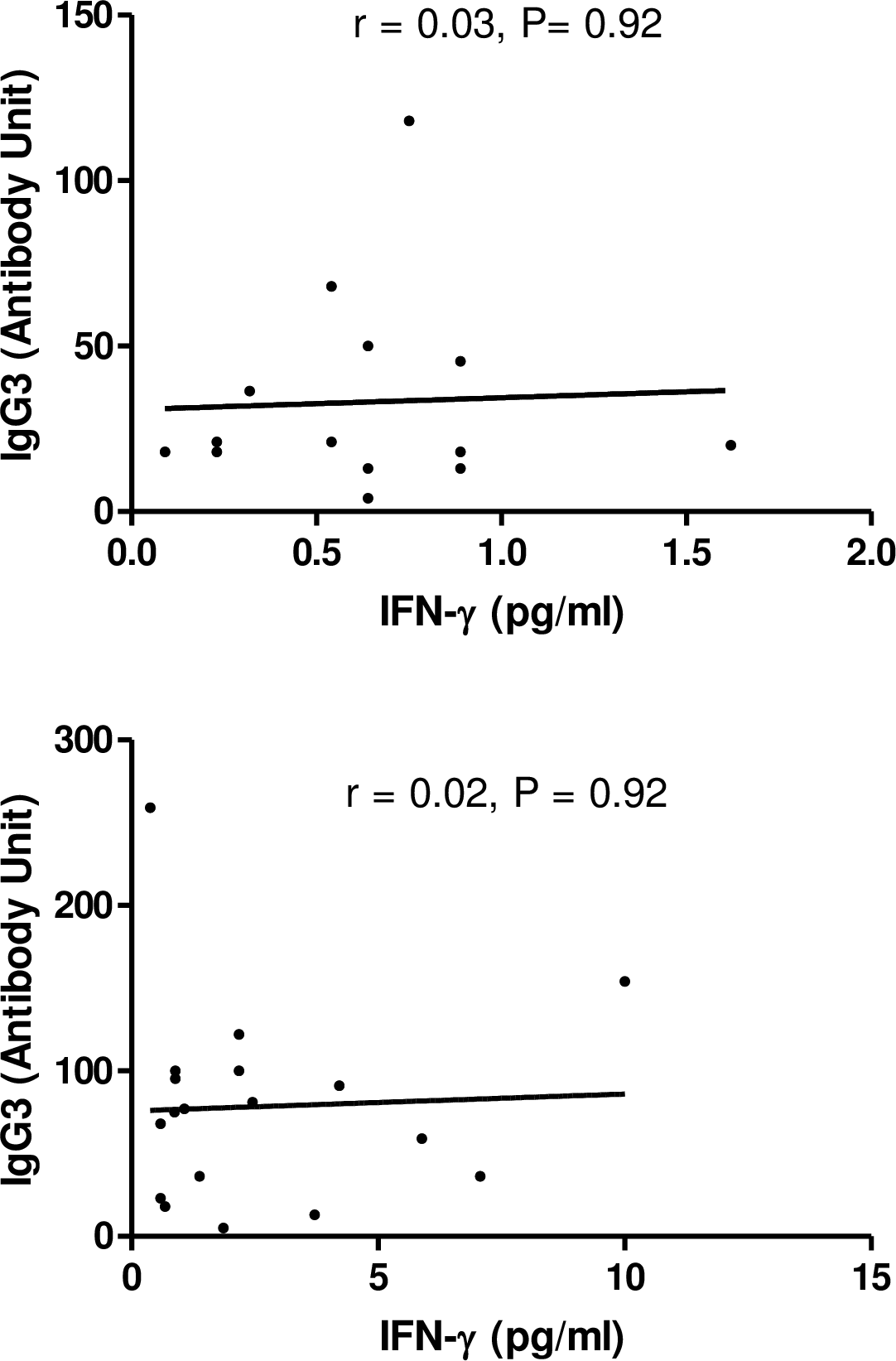

Supplement: S1 Fig — Correlation between IFN-γ with filarial specific IgG3 in infected (A) and infection free (B) children born to infected mother. (TIF) [file pntd.0006824.s002.tif]
